# Alzheimer's Disease and Cardiovascular Disease: A Particular Association

**DOI:** 10.1155/2020/2617970

**Published:** 2020-05-05

**Authors:** Giacomo Tini, Riccardo Scagliola, Fiammetta Monacelli, Giovanni La Malfa, Italo Porto, Claudio Brunelli, Gian Marco Rosa

**Affiliations:** ^1^Department of Internal Medicine (DIMI) Clinic of Cardiovascular Diseases, University of Genoa, Genoa, Italy; ^2^Department of Internal Medicine (DIMI) Clinic of Geriatric Diseases, University of Genoa, Genoa, Italy; ^3^IRCCS Ospedale Policlinico San Martino, Italian IRCCS Cardiovascular Network, Genoa, Italy

## Abstract

**Methods:**

This review is based on the material obtained via MEDLINE (PubMed), EMBASE, and Clinical Trials databases, from January 1980 until May 2019. The search term used was “Alzheimer's disease,” combined with “cardiovascular disease,” “hypertension,” “dyslipidaemia,” “diabetes mellitus,” “atrial fibrillation,” “coronary artery disease,” “heart valve disease,” and “heart failure.” Out of the 1,328 papers initially retrieved, 431 duplicates and 216 records in languages other than English were removed. Among the 681 remaining studies, 98 were included in our research material on the basis of the following inclusion criteria: (a) the community-based studies; (b) using standardized diagnostic criteria; (c) reporting raw prevalence data; (d) with separate reported data for sex and age classes.

**Results:**

While AD and CVD alone may be considered deleterious to health, the study of their combination constitutes a clinical challenge. Further research will help to clarify the real impact of vascular factors on these diseases. It may be hypothesized that there are various mechanisms underlying the association between AD and CVD, the main ones being hypoperfusion and emboli, atherosclerosis, and the fact that, in both the heart and brain of AD patients, amyloid deposits may be present, thus causing damage to these organs.

**Conclusions:**

AD and CVD are frequently associated. Further studies are needed in order to understand the effect of CVD and its risk factors on AD in order to better comprehend the effects of subclinical and clinical CVD on the brain. Finally, we need to clarify the impact of the underlying hypothesized mechanisms of this association and to investigate gender issues.

## 1. Introduction

During ageing, the prevalence of dementia doubles every 4 to 5 years after the age of 60 years. Indeed, more than 30% of subjects over 80 years old are likely to suffer from dementia [1]. Epidemiological data indicate that the most common cause of dementia in the elderly is Alzheimer's disease (AD) [2]. Furthermore, these data suggest that cardiovascular disease (CVD) and cardiovascular risk factors are associated with an increased risk of AD and its precursor clinical stage: mild cognitive impairment (MCI).

In this regard, clinical studies have indicated that CVD and dementia share similar genetic and biochemical profiles and common triggers [3–5]. The principal relevance of these associations lies in the fact that they reveal a potential opportunity to prevent dementia through the management and treatment of CVD and its risk factors by means of both pharmacological therapy (promising examples from antihypertension medication trials) and lifestyle modifications aimed at improving cardiovascular health [6]. This review investigates the relationship between AD and CVD and its risk factors, with a view to explaining the underlying mechanisms of this association.

## 2. Methodology

A narrative review was performed by following the standard methods of the Cochrane Collaboration and PRISMA declaration. The material searched was obtained by the following search engines: MEDLINE (PubMed), EMBASE, and Clinical Trials databases, from January 1980 until May 2019. The search terms used were “dementia” and “Alzheimer's disease” combined with “cardiovascular disease,” “hypertension,” “dyslipidaemia,” “diabetes mellitus,” “atrial fibrillation,” “coronary artery disease,” “heart valve disease,” and “heart failure.”

Out of the 1,328 papers initially retrieved, 431 duplicates and 216 records in languages other than English were removed. Among the 681 remaining studies, 98 were included in our research material on the basis of the following inclusion criteria: (a) the community-based studies; (b) using standardized diagnostic criteria; (c) reporting raw prevalence data; (d) with separate reported data for sex and age classes.

## 3. Mechanisms of Cardiovascular Involvement in Alzheimer's Disease

A potential “head-to-heart” link may be hypothesized in AD. This link has been ascribed to a reduction in cerebral perfusion. As the brain is highly vascularized, receiving 15% of cardiac output and consuming about 20% of the body's total oxygen supply, it is particularly vulnerable to the impairment of cerebral perfusion, which is a frequent event in heart failure (HF) [7], particularly in the forms due to reduced systolic function. Cerebral hypoperfusion has been supposed to actively contribute in the formation of tau-containing neurofibrillary tangles and amyloid *β* (Aβ) plaques which characterize AD, although, to date, data from human subjects confirming this hypothesis are lacking [8, 9]. Cerebral hypoperfusion causes a metabolic energy crisis of the brain cells, thereby leading to acidosis and oxidative stress [5]. An acid environment stimulates activation of lysosomal enzymes, thus leading to the hyperphosphorylation of tau proteins. These hyperphosphorylated tau proteins cluster and give rise to so-called neurofibrillary tangles [10].

Furthermore, the altered metabolism of the neurons causes upregulation of beta-secretase 1, a protease which is responsible of the cleavage of the amyloid precursor proteins, thereby determining accumulation of A*β* and the formation of amyloid plaques [11]. Another mechanism which favours the formation of amyloid plaques, in cases of hypoperfusion, is the breakdown of the blood-brain barrier, which impairs the clearance of A*β* [12].

Furthermore, both in the heart and in the brain of AD patients, amyloid and/or atherosclerotic angiopathy may be found, causing damage to these organs [13, 14]. It may be hypothesized that A*β* may be deposited in the distal organs and vessels, such as the skeletal muscle, skin, kidneys, lungs, and intestine, through the bloodstream, causing other forms of nonneurological amyloid pathology. Indeed, deposits of A*β* may be found both in the myocardium and in the brain. In this regard, previous studies have reported the presence of protein deposits in the myocardium of patients with idiopathic dilated cardiomyopathy (iDCM) and have ascertained that such deposits are of the same type as those found in AD, thus giving rise to the idea of amyloid pathology as a multiple organ syndrome [15, 16]. Genetics also seems to play an important role, in that genetic variants of the same gene associated with early-onset AD have been observed in familial [17] and sporadic cases of iDCM [15].

In a retrospective, cross-sectional study, Troncone et al. found that AD patients presented compromised myocardial function and intramyocardial deposits of A*β*. Echocardiographic measurements of myocardial function suggested that patients with AD present early diastolic dysfunction, while proteomic approaches have been used to identify and quantify A*β* deposits in AD heart and brain specimens. Indeed, as in the brain, two forms of A*β* (A*β* 42 and A*β* 40) are present in the heart, and their expression is increased in AD [18].

AD is closely correlated with age. Indeed, reduced diastolic myocardial function is a normal event in cardiac aging. However, AD patients were shown to display worse diastolic function at younger ages compared to controls. Additionally, it was found that neither hypertrophy nor aortic valve stenosis was the underlying cause of the diastolic dysfunction observed in younger AD patients. Probably, other mechanisms, such as accumulation of A*β* in the myocardium, are involved. Troncone's study was the first report of both compromised myocardial function and intramyocardial deposits of A*β* in AD patients. Thus, a novel biological scenario emerges in which AD may be seen either as a systemic disease or as a metastatic disorder leading to HF.

## 4. Role of Cardiovascular Risk Factors in Alzheimer's Disease

The most intriguing aspect of the association between cardiovascular risk factors and AD occurrence is the timing of this association. The case of arterial hypertension is typical. While late-life hypertension does not correlate with any dementia, several analyses have shown how midlife hypertension is associated with dementia and AD [19, 20]. However, probably because of an expression of hypoperfusion, late-life low blood pressure may be associated with dementia [19].

Midlife hypertension negatively impacts on cognitive function in both men and women, independently from other cardiovascular risk factors, and raises the risk for late-life dementia [20, 21]. This association was found also in the Framingham cohort and was even stronger in those individuals whose hypertension was untreated [21]. What is interesting, however, is that while hypertension exacerbates the risk of late-life dementia, it is also associated with early signs of MCI [22]. A definite causative mechanism underlying the relationship between hypertension and dementia, and particularly AD, has not yet been found. It is, however, conceivable that long-standing hypertension, being closely related with endothelial dysfunction, arterial stiffness, and atherosclerosis, is linked with cerebral hypoperfusion [21, 23].

Moreover, as indicated by the alterations found in hypertensive patients suffering from stroke, hypertension causes cerebral microinfarcts, lacunar infarcts, macrobleedings, and microbleedings [24]. These phenomena are closely related to cognitive dysfunction in general and also to AD [20]. Furthermore, hypertension also induces white matter alterations and is related to the abnormal accumulation of A*β*, which are typical features of AD [20, 23].

Finally, animal models have shown that hypertension causes blood-brain barrier disruption [25] and influences A*β* linked-gene expression in the hippocampus [26]. As said, it is not clear whether hypertension is directly involved in the genesis of AD or whether the alterations which it induces constitute a substrate for AD genesis and/or development [24].

Most importantly, it seems that antihypertensive treatments may reduce the occurrence of dementia.

Although there are no conclusive results to indicate that specific classes of antihypertensive drugs act against dementia [23, 27], a recent study has shown that the more intense the antihypertensive treatment is, the lower is the risk for developing dementia [27].

As well as for hypertension, longitudinal studies have shown a strong association between midlife high total cholesterol and AD [28]. Furthermore, hyperlipidaemia may cause carotid atherosclerosis which may be associated to AD [29]. Moreover, AD patients tend to have lower HDL levels and higher LDL levels than matched controls. Of all cardiovascular risk factors, dyslipidaemia is the easiest to link with AD. Indeed, apolipoprotein E (ApoE), the allele variability of which is implicated in AD susceptibility, is involved in lipid transport and metabolism [30]. Moreover, carriers of ApoE4, the form linked with AD, usually have higher cholesterol levels [19]. Cholesterol levels, both brain storage and plasma levels, are thought to influence the activity of the brain's secretase enzymes. In brief, higher levels of cholesterol and its oxidative metabolites are associated with increased A*β* production [18, 28–30] whilst lower levels are associated with reduced amyloidogenesis. Evidence of associations between cholesterol and A*β* levels is also suggested by the fact that, in several studies, the use of statins has been seen to correlate with a decreased occurrence of AD [19, 28]. However, the way in which statins lower the risk of AD is not clear. Although a clear correlation with lower levels of brain cholesterol has been reported, the fact that statins with different abilities to penetrate the blood-brain barrier induce a similar reduction in AD prevalence suggests that other mechanisms are involved [28].

Finally, both type 2 diabetes mellitus (DM2) and a prediabetic status are also known to raise the risk for dementia in general and also of vascular dementia and AD [31]. Many mechanisms are considered to be responsible for this association. As in hypertension, the role of endothelial dysfunction in DM2 and in the atherosclerotic process is well known. DM2 also causes cerebral microvascular damage [19, 31]. Another potential mechanism is neurotoxicity caused by excessive blood glucose levels. In addition to all these aspects, which are common to all types of dementia, there is specific evidence that link DM2 to AD. Indeed, insulin is involved in A*β* clearance [19]. The insulin-degrading enzyme (IDE) can metabolize several molecules apart from insulin itself, including A*β* [30, 31].

It has been suggested that high levels of insulin, as in the hyperinsulinemic state typical of DM2, may deviate IDE action. In other words, with more insulin to degrade, IDE cannot act on A*β*, and therefore its levels increase [19, 31]. Other risk factors, including smoking, obesity, and sedentary lifestyle, are associated with the development of both AD and CVD [32, 33].

With reference to the relationship between gender differences and cardiovascular risk factors for AD, while a diagnosis of hypertension, high cholesterol, and diabetes has been associated with a greater risk of developing both CVD and AD in both women and men, women with these risk factors seem to be at greater risk of AD than men [34]. Conversely, smoking, the presence of coronary atherosclerotic disease, and brain injury with loss of consciousness are more predisposing in male sex [35].

## 5. Modifiable Protective Factors in Alzheimer's Disease

Various protective factors can be identified, including physical activity, higher education, occupation, current cognitive activity, and efficacious treatment of vascular risk factors. Physical activity has been demonstrated to have positive effects on cognition and may play a role in AD prevention [36, 37]. Having a higher education/occupation and greater engagement in cognitive activities provides a higher reserve against AD [38].

In this regard, the Finnish Geriatric Intervention Study to Prevent Cognitive Impairment and Disability (FINGER study) [39] suggested that diet, exercise, cognitive training, and vascular risk monitoring could improve or maintain cognitive function in older patients at risk of dementia. However, the transferability of these results to people who already have dementia should be examined in further prospective studies.

Finally, an adequate control of risk factors may be protective. In a meta-analysis of observational studies, Wong et al. showed that statins could provide a slight benefit in the prevention of AD and all-type dementia. This benefit should, however, be interpreted with caution, owing to the fact that observational studies are subject to bias, and if a well-designed randomized controlled trial is conducted, the benefit observed may no longer be present [40].

In another review, Geifman and colleagues analyzed datasets of clinical trials and the results of prospective observational studies. They found that the use of statins may be useful in all AD patients and displayed potentially greater therapeutic efficacy in those homozygous for ApoE4 [41]. A better cognitive performance was found in statin users, who, moreover, presented a lower incidence of AD.

## 6. Cardiovascular Involvement in Alzheimer's Disease

### 6.1. Atrial Fibrillation and Dementia

Atrial fibrillation (AF) is the most common cardiac arrhythmia and has been associated with the occurrence of clinical or silent cardioembolic stroke (CES), leading to dementia [42]. In support of this hypothesis, a meta-analysis showed a close relationship between AF and a higher risk of dementia, though it was restricted to individuals with stroke [43]. By contrast, another study found that stroke-free individuals with AF performed worse on memory and learning tasks and had a reduced hippocampal volume [44].

Both memory function and hippocampal volume are strongly related to AD; this suggests that there might be additional pathways that explain the association between AF and AD [45]. It has been hypothesized that AF might also cause dementia through cerebral hypoperfusion. A low cardiac output may induce cerebral hypoperfusion, even if transient, thereby determining vascular dementia [46], regardless of whether AF leads to ischemic stroke. Cerebral hypoperfusion causes damage to nerve cells, thereby contributing to the aetiology of AD [47]. In addition, cerebral hypoperfusion may accelerate the three major pathological hallmarks of AD, namely, senile plaques (A*β* 42), cerebral amyloid angiopathy (A*β* 40), and neurofibrillary tangles (phosphorylated tau) [48].

The presence of neurofibrillary tangles may also be indirectly caused by an increase in amyloid, as after a long period of time, both an increase in A*β* production and failure in A*β* clearance may cause tau phosphorylation [49]. Moreover, cerebral ischemia may be induced by both forms of AF: permanent and nonpermanent. The crucial difference between these two forms is the reduction in cardiac output in permanent AF, which increases the likelihood of cerebral hypoperfusion [50]. Vital organs, such as the brain, present a mechanism of autoregulation to support circulation, even if cardiac output decreases [51]. However, in the presence of long-term AF, this compensatory mechanism fails, thereby causing a decrease in cerebral circulation [52]. This datum may, at least partially, explain the correlation between AF and AD, even in younger patients with a presumably intact cerebral autoregulation system. In a paper by Dublin et al. [48], when permanent AF was observed, the relative risks of the three main pathological hallmarks of AD, namely, senile plaques, neurofibrillary tangles, and cerebral amyloid angiopathy, were 1.47, 1.40, and 1.50, respectively, in comparison with control subjects.

Conversely, in the case of nonpermanent AF, the corresponding relative risks were reduced to 0.82, 0.42, and 0.74, respectively. The lack of significant differences may be due to the limited number of cases autopsied. However, this study sheds light on the mechanisms linking AF and AD. In the same study, the relative risk of cerebral infarction was 1.84 in permanent AF and 1.81 in nonpermanent AF, with no apparent difference, making it unlikely that CES caused by AF mediates the difference. A discrepancy regarding the AF-AD link can be found between epidemiological studies [53–58] and neuropathological studies [48, 59–61]. While epidemiological studies have suggested an association between AF and AD, especially in younger individuals, neuropathological studies, including autopsies, have not found this link. In clinical studies, the contribution of AF to AD pathogenesis may be obscured in the elderly because of the age-dependent, absolute increase in AD prevalence, regardless of AF [53]. The discrepancy between epidemiological and neuropathological studies may be attributed to the fact that, in the latter, AF may be not properly diagnosed, owing to the lack of adequate monitoring.

Furthermore, with the exception of the study by Dublin [48], these studies do not typically distinguish persistent AF from nonpersistent AF when taking medical history and performing examination. On the other hand, in epidemiological studies, a multitude of possible confounders, such as comorbidities of AF, may underlie the link between AF and AD [53]. In the presence of AF, another aspect which must be taken into account is whether rate-control is a promising strategy in AF in terms of reducing the incidence of AD. In a paper by Cacciatore and coworkers, the effects of ventricular response in AF and cognitive impairment were monitored for 10 years in 44 AF and 314 non-AF patients whose Mini-Mental State Examination (MMSE) scores were less than 24. Those who had AF with low/high ventricular rate (average rate <50 or ≥90 beats per minute) showed a higher rate of MCI than those with AF plus moderate ventricular rate (50< beats per minute <90) [59]. AF was a strong predictor of dementia (HR = 4.10) independently of other factors such as age, sex, and heart rate. Moreover, the low/high ventricular response was predictive of dementia in the presence (HR = 7.70), but not in the absence (HR = 1.85), of AF in the cognitively impaired elderly subjects. Interestingly, vascular dementia was more frequent in the presence than in the absence of AF (57.0 versus 46.0%, *p* < 0.01) although subjects with stroke were excluded from the analysis; this suggests that hypoperfusion is the prevalent mechanism underlying AD in AF patients with low/high ventricular rate.

Another issue which must be taken into account in the presence of AF is the adoption of a rate-control vs. rhythm-control strategy. For what concerns cognitive status, a substudy of the AFFIRM (Atrial Fibrillation Follow-Up Investigation of Rhythm Management) trial showed that both strategies have the same impact on cognitive status. Indeed, in 245 subjects, the average MMSE scores at the baseline and after a mean follow-up of 3.6 years did not show significant differences [60]. Unfortunately, however, the findings of this may be considered to have been weakened by the small sample size. Multiple observational studies with long-term follow-up and large-scale randomized controlled trials with comprehensive neurocognitive assessment and brain imaging are needed in order to clarify this issue. In another study, which enrolled 17 patients with medically refractory rapidly-conducted AF who underwent ablation (average age, 55.3 years) followed by pacemaker implantation, left ventricular systolic function, brain perfusion, and cognitive function improved 3 months after the operation, implying that a combination of ablation plus pacing holds promise of cognitive recovery [61].

However, further studies are needed in order to confirm these results in AD pathology. A further subject of investigation is the impact of anticoagulant therapy on the occurrence of dementia in AF patients. In this regard, a recent retrospective registry study of 444,106 patients with a hospital diagnosis of AF showed that patients on anticoagulant treatment at the baseline had a significantly lower risk of incident dementia than patients without anticoagulant treatment (HR 0.71; 95% CI, 0.68–0.74) [62].

Furthermore, in this study, a direct comparison between nonvitamin K antagonist oral anticoagulants (NOACs) and vitamin K antagonist (warfarin) showed no difference in terms of the lowered risk of dementia (HR 0.97; 95% CI, 0.67–1.40).

On the other hand, in an observational study, 5,254 patients aged 18 years or more who started anticoagulation therapy (NOACs or warfarin) were examined with regard to the composite outcome of dementia/stroke/transient ischemic attack; NOACs proved superior to warfarin for the composite outcome [63].

This study also suggested that the dementia-free survival rate was significantly higher in NOAC-treated subjects than in those on warfarin (*p*=0.02).

However, dementia subtypes were not described, which may be considered a limitation of the study. Further research is needed in order to ascertain the incidence of dementia in patients receiving either NOACs or warfarin and to evaluate whether warfarin and NOACs are effective in preventing the onset and progression of AD.

### 6.2. Coronary Artery Disease in Alzheimer's Disease

An association between coronary artery disease (CAD) and coronary calcifications, a marker of atherosclerotic burden with white matter lesions (WML) and grey matter (GM) changes, has been demonstrated by previous studies [64, 65].

In addition, the prospective Rotterdam Study has shown that patients with severe arteriosclerosis carry a two-to three-fold higher risk of developing either AD or all-cause dementia than those without arteriosclerosis [66]. These data were confirmed by the Ageing, Cognition, and Dementia (AgeCoDe) in primary care patients study, which also showed that CAD has a deleterious effect on MCI after patients receive a diagnosis of AD [67]. The study underscored the key role of cardiovascular prevention and highlighted its benefit on cognitive impairment in dementia. Aronson et al. investigated the relationship between dementia and CAD in 69 elderly patients at risk of dementia, who participated in the Cardiovascular Risk Factors, Aging, and Dementia (CAIDE) study. CAD (particularly of longer duration) was related to lower cortical thickness and GM volume (on magnetic resonance imaging (MRI)) [68]. This association was seen to be influenced by blood pressure (BP) in that people with CAD and midlife hypertension and also those with CAD and declining BP appeared to be at greater risk of GM atrophy [69]. Indeed, carriers of the ApoE4 allele have shown to present a more marked association between the extent of CAD and the density of cardinal neuropathological lesions of AD than subjects who do not present this allele [68].

It has been seen that several cardiovascular risk factors, such as hypertension [70], cholesterol [71], and insulin resistance [69], may interact with ApoE, thereby increasing the incidence of AD and accelerating its evolution. Thus, ApoE might impact on both CAD and AD neuropathology. However, on controlling for ApoE genotype in the entire sample, significant relationships emerged between CAD and neuropathological lesions, which may be considered the hallmarks of AD [72]. This suggested that, although the ApoE genotype significantly contributes to this association, a link between CAD and AD may be present regardless of this genotype. Finally, it must be taken into account that genetic testing for ApoE, which is mainly conducted in research settings and is not yet widely available, cannot predict whether individuals will develop Alzheimer's disease, but only whether they may be more likely than others to do so; its clinical utility is therefore limited. Furthermore, in the literature, few studies have indicated a link between myocardial infarction (MI) and dementia [73, 74].

In a population-based cohort study, Ikram et al. showed that men with unrecognized MI have an increased risk of stroke [73]. Furthermore, a cross-sectional evaluation in the Rotterdam study showed a positive association between prior MI and cognitive impairment due to brain hypoperfusion [66]. Another analysis provided by Ikram and coworkers demonstrated that unrecognized MI was associated with an increased risk of dementia, increased WML, and brain infarcts, thus supporting the possibility that small vessel disease may be one mechanism by which the risk of dementia increases in MI subjects [74].

In addition, the Bronx Aging Study showed that women with a history of MI had a fivefold increase in the risk of dementia [68].

Gharacholou et al. showed that older subjects with prior MI had measurable cognitive impairment prior to dementia [75]. Probably MI subjects and AD subjects also share a genetic background, involving abnormalities in cholesterol metabolism and an upregulation of inflammation, as atherosclerosis plays an important role in both CAD and AD.

This hypothesis is corroborated by findings from the Cardiovascular Health Study, which showed that peripheral artery disease, another manifestation of arteriosclerosis, was also strongly associated with an increased risk of AD [76].

Other mechanisms which might be common to AD and CAD include diminished cardiac function, hypoperfusion, and emboli [47, 77]. However, the impact of MI on dementia remains controversial, and findings lack consistency. Studies, such as the Honolulu-Asia Aging Study found no association between MI and later cognitive impairment [78].

Moreover, in a population-based study, Bursi and colleagues found that the frequency of MI preceding dementia was identical in the index cases and in control subjects, thus showing no significant evidence of a positive association between dementia and MI [79].

Further research is needed in order to improve our understanding of the influence of MI on subsequent dementia.

Another issue which must be investigated is the fact that the Rotterdam study showed that unrecognized MI was associated with the risk of AD, whereas recognized MI was not.

Furthermore, it must be taken into account that cholinesterase inhibitors, which constitute the first-line symptomatic therapy for AD, may be useful in ischemic HF after MI. Arikawa et al. [80] demonstrated that Donepezil, a cholinesterase inhibitor, suppressed cardiac remodelling in a mouse model of ischemic HF after MI, owing to its anti-inflammatory properties. In this regard, Monacelli and Rosa had previously shown that cholinesterase inhibitors exert cardioprotective effects apart from cholinesterase inhibition, thus profiling a new line of therapeutic use for these drugs [81].

### 6.3. Heart Valve Disease

In the literature, autoptic studies have revealed significant aortic and mitral valvulopathies in AD subjects in comparison with nondemented controls [82].

In a study by Boudoulas and coworkers, left atrial dysfunction, which appears in the presence of chronic mitral valve disease, has been shown to contribute to AF and thereby increases the risk of dementia [83].

These data were confirmed by Rodriguez et al. in a community-based study of 2,680 subjects enrolled in the Cardiovascular Health Study [84]. In this analysis, participants, without a history of stroke, but with mitral valve calcification had a 33% greater risk of covert brain infarct on MRI analysis.

Other studies have revealed the presence of brain infarcts in association with calcification of the aortic valve; this supports the association between valve disease and a higher risk of stroke and MCI [85].

Thus, evaluating patients for aortic and mitral valve disease may play an important role in the workup of dementia and its prevention.

### 6.4. Heart Failure

HF is a complex clinical syndrome characterized by the fact that the heart is unable to adequately supply blood to other organs, particularly frequent in the elderly (≥65 years of age); indeed, 6–10% of these subjects have a diagnosis of HF [86].

In various studies, it has been reported that this clinical syndrome may be associated with cognitive impairment and dementia [87, 88]. In a pilot case-control study, Beer et al. found that HF patients had lower scores than controls on neuropsychological tests designed to judge cognitive functions for the diagnosis of dementia [89].

In a Swedish study, a relationship between HF and dementia, particularly AD, was found. It was also demonstrated that antihypertensive drugs slightly reduced the risk of developing this pathology [90]. In addition, the Framingham Offspring Study demonstrated that a decreased myocardial function was related to lower brain volume (a hallmark of dementia), even in the absence of overt HF [91].

The pathophysiological mechanism that underlies the association between HF and cognitive impairment and dementia is still being investigated. In HF, low cardiac output, combined with impaired cerebral autoregulatory mechanisms, may result in decreased cerebral blood flow, leading to hypoxia and damage to nerve cells [5, 92]. Thus, cerebral hypoperfusion leads to cognitive impairment and dementia [46].

Furthermore, it has been shown that in patients who undergo heart transplantation, cerebral blood flow is restored and cognitive performance improves [93].

**Figure 1:**
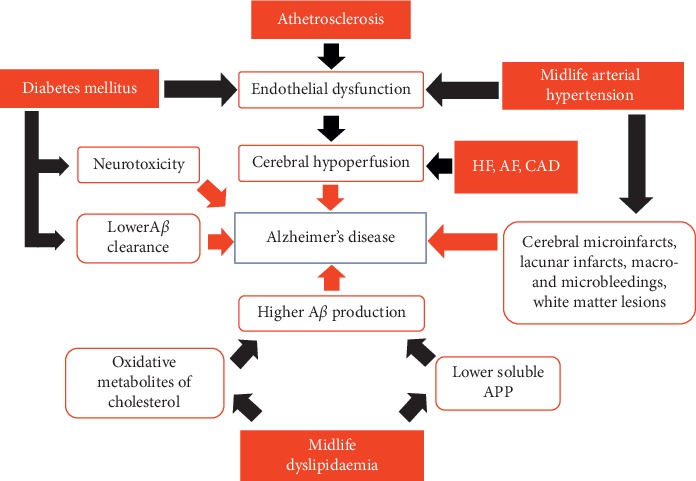
Interplay between cardiovascular risk factors and increased risk of mild cognitive impairment and Alzheimer's disease. A*β*, amyloid *β*; AF, atrial fibrillation; APP, amyloid precursor protein; CAD, coronary artery disease; HF, heart failure.

Additionally, HF increases the risk of emboli and microvascular pathology, such as white matter lesions and lacunae, which are, in turn, related to an increased risk of dementia [76, 94] (Figure 1).

## 7. Conclusions

While AD and CVD alone are currently regarded as health threats, the study of their combination constitutes a clinical challenge that requires an interdisciplinary approach to patient management and treatment.

In this review, the authors have illustrated the data available in the literature on the relationship between AD and CVD and its risk factors and have investigated the underlying mechanisms of this association. To conclude, the effect of CVD and cardiovascular risk factors on AD remains an active area of research. Cardiovascular prevention trials will help to establish whether vascular factors have a clear impact on this pathology and to clarify the effects of subclinical CVD on the brain. Specifically, the ApoE genotype is thought to play a relevant role. Indeed, carriers of the ApoE4 allele display a more marked association between the extent of CAD and the density of cardinal neuropathological lesions of AD than subjects who do not carry this allele. This relationship, which appears to be stronger in women, nevertheless needs to be confirmed. Many questions concerning the underlying mechanisms of the relationship between AD and CVD remain unanswered. Undoubtedly, hypoperfusion and emboli, which are hallmarks of HF (associated to reduced systolic function) may constitute a link between AD and CVD. These mechanisms certainly also have a role in the interaction between AF and AD. However, as clinical and neuropathological studies are discordant as to whether there is a link between these two diseases, further studies will be needed in order to clarify this relationship.

Another important mechanism may be constituted by the fact that amyloid deposits may be found in both the heart and brain of AD patients, causing damage to these organs, especially diastolic dysfunction in the heart (HF with preserved systolic function, typical of ageing).

Finally, it may be hypothesized that another underlying mechanism is the association between white and grey matter lesions and coronary atherosclerosis, and even valvulopathies (probably through an embolic mechanism) may be associated to AD. Another important public health question is how AD and CVD intersect in the oldest-old, as the population ages. For instance, in the USA, the population of adults aged ≥90 years is expected to grow more than six-fold by 2050 [95]. As the average lifespan increases, the social and economic consequences of AD and CVD are expected to expand accordingly. However, owing to the difficulty of recruiting and diagnosing the oldest-old, very few studies have examined the relationship between AD and CVD in this population. A review by Gardner et al. examined the question of the interaction of CVD and AD in the oldest-old [96]. While the role of traditional risk factors in these subjects needs to be further investigated, improved diagnostic criteria and neuropsychological norms are required in order to obtain an accurate diagnosis. Conversely, no drug trials have as yet focused specifically on dementia in the oldest-old. Indeed, these subjects may develop adverse side effects more easily than younger patients; consequently, targeted therapies may be less effective and only partially undertaken. Undoubtedly, establishing and maintaining a healthy active lifestyle from midlife onwards appear to be of paramount importance for what regards keeping dementia at bay in the oldest-old [97].

Future population-based studies should therefore aim to include this cohort in order to better clarify the nature of disease interactions and to identify preventive measures for reducing cardiovascular risk in this specific population. Indeed, we need more accurate models for assessing prognosis and life expectancy in multimorbid older adults with AD and CVD.

To conclude, when we speak of AD and CVD, another problem which must be taken into account is the presence of gender issues. Gender differences exist in the incidence of AD, in that women are at higher risk of AD, and women with a history of HF are more likely to develop cognitive impairment and loss of memory than those without HF [98]. Furthermore, the impact of the various cardiovascular risk factors on the occurrence of AD may differ between men and women [99].
